# Nutritional Composition, Chemical Safety, and Microbiological Quality of Farmed Palm Weevil Larvae (*Rhynchophorus phoenicis*) From a Commercial Rearing Unit in Ghana

**DOI:** 10.1002/fsn3.72402

**Published:** 2026-09-27

**Authors:** Benjamin Kumah Mintah, Hilary Kwesi Ketemepi, Ebenezer Asiamah, Akwasi Akomeah Agyekum, Ethel Juliet Serwa Blessie, Akua Boatemaa Arthur, Mokhtar Dabbour

**Affiliations:** ^1^ CSIR‐Food Research Institute Accra Ghana; ^2^ Department of Agro‐Processing Technology and Food Bio‐Sciences CSIR College of Science and Technology (CCST) Accra Ghana; ^3^ Nutrition Research Centre Ghana Atomic Energy Commission Accra Ghana; ^4^ Department of Agricultural and Biosystems Engineering, Faculty of Agriculture Benha University Moshtohor Egypt

**Keywords:** edible insects, health risk assessment, heavy metal safety, microbiological safety, nutritional composition, *Rhynchophorus phoenicis*, UV–Vis spectroscopy

## Abstract

The consumption of palm weevil larvae (*Rhynchophorus phoenicis*), a traditional edible insect in Ghana, is on the rise; nevertheless, comprehensive data are scarce regarding the impact of culinary processing on its nutritional profile and safety. This research assessed the proximate composition, mineral content, chemical safety (heavy metals), health risk, microbiological quality, and physicochemical properties of fresh, boiled, fried, and roasted *R. phoenicis* larvae. The proximate composition was analyzed using AOAC methods, essential and harmful metals were measured through AAS and ICP‐MS, microbiological quality was evaluated via standard culture methods, and potential health risks were determined using estimated daily intake (EDI) and target hazard quotient (THQ). Frying and roasting significantly reduced moisture content (to 30.5% and 46.2%, respectively) and increased protein content by 133.36% and 114.89%, respectively, compared to fresh larvae. Fat content was highest in fried samples (34.4%), resulting in elevated energy values (442.19 kcal/100 g). Calcium and iron contents increased significantly following frying and roasting, while zinc was better retained in fresh and boiled samples. Mercury and arsenic were below detection limits in all samples, whereas lead (0.33–0.39 mg/kg) and cadmium (0.82–0.94 mg/kg) exceeded Codex maximum limits; nevertheless, THQ values for all metals were < 1, indicating low non‐carcinogenic health risk. ILCR values for cadmium ranged from 3.4–4.1 × 10^−6^, slightly above the US EPA acceptable risk level (1 × 10^−6^). MOE values for lead ranged from 1.79–1.96, indicating that exposure from PWL consumption is within a range of concern under the EFSA framework. Consuming fresh PWL unwashed (APC = 7.0 log_10_ CFU/g) or washed with water only (APC = 5.2 log_10_ CFU/g) is not recommended based on microbiological safety standards. UV‐spectra data corroborated the study findings.

## Graphical abstract

Frying and roasting significantly reduced moisture content and increased protein content of *Rhynchophorus phoenicis* larvae. Fat content was highest in fried samples, resulting in elevated energy values. Calcium and iron contents increased significantly following frying and roasting. THQ values for heavy metals were < 1, representing low non‐carcinogenic health risk. ILCR values for cadmium were slightly above the US EPA acceptable risk level. Unwashed larvae or those washed with just water are not recommended for consumption, as per existing microbiological safety standards.

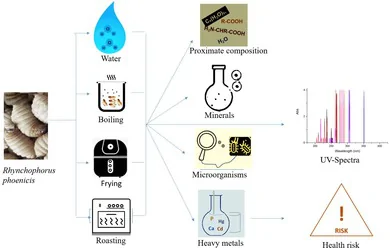

## 1 Introduction

The consumption of insects (entomophagy) is receiving attention globally due to environmental pressures, food insecurity, population growth, and the rising cost of rearing animals and their protein thereof (van Huis et al. 2013). Their consumption is traditionally accepted in several (≈113) countries, where they are primarily collected from their natural environment (wild) (Dobermann et al. 2017); but, with the increasing attention on and demand for insect‐based foods worldwide, many enterprises have started intensive farming systems (Wade and Hoelle 2020). Compared to conventional livestock production, edible insects present several benefits to humans and the environment: reduce land and water usage, have lower organic waste (feed) conversion ratio, reduce pesticide usage in agro‐crop production and the associated decrease in financial burden on farmers (Cerritos 2009; Cerritos and Cano‐Santana 2008), and have lower global warming potential (Gebreyes and A.Teka 2025; van Huis et al. 2013). Further, their rich nutritional profile (Mintah et al. 2020) makes them attractive in human nutrition. In this light, researchers and other stakeholders are promoting consumption of insects and insect‐based foods by processing and/or concealing appearance through novel product formulations (Navina et al. 2025; Mintah et al. 2019; van Huis et al. 2013). In Ghana (particularly in the South) is a popular edible insect called “Akokono”—palm weevil larvae (PWL, *Rhynchophorus phoenicis*). This insect (presently farmed) is not only considered a delicacy, but it is also sold on the market; thus, giving it a commercial or economic value. That is, it is a source of livelihood and nutrition for many households in the regions (Anankware et al. 2016; Raheem et al. 2018).

In culinary, *Akokono* (PWL) is normally processed/prepared for consumption (at home, specialty stores, or on‐street) by either boiling, frying, and/or roasting. Even though it is already on the market or being consumed in boiled, fried, and roasted form, there are, until now, no particular standards, guidelines, or regulations regarding potential hazards (microbiological, physical, or chemical) established in Ghana (likewise other parts of the world) for insects as food or insect‐based food products. By general inference, edible insects produced for human consumption or available to Ghanaian/global consumers must meet the same safety and hygiene standards as other foods available in Ghana and other regions of the world. It is also unclear as to how the mentioned heat treatments (boiling, frying, and roasting) affect the proximal and micronutrient composition of *Akokono*. This study therefore sought to examine the microbial and chemical safety (risk‐based) of 4 culinary‐treated (fresh/washed, boiled, fried, and roasted) *Akokono*; and as well examined the influence of the said treatments on the proximal and micronutrient composition of the sample(s); as such data are imperative in the estimation of potential risk of malnutrition and/or human health risks. Thus, the outcome provides valuable insights into the nutritional value and potential safety considerations of consuming *Akokono* in Ghana. Further, the information can help policymakers, food safety authorities, and consumers make informed decisions regarding the inclusion of edible insects (*Akokono*) in the local diet and promote their sustainable use as a nutritious food source.

## 2 Materials and Methods

### 2.1 Samples and Chemicals

*R. phoenicis* larvae (PWL) samples acquired from a commercial farm (in Kumasi, Ghana) were used for the study. Samples used were from the same batch. All reagents and chemicals used were of analytical grade. Certified reference materials (CRM: DORM‐4, Fish Protein Certified Reference Material for Trace Metals), standard stock solutions (1000 mg/L for Pb, Cd, Hg, As), nitric acid (HNO_3_, 65%), and hydrogen peroxide (H_2_O_2_, 30%) were sourced from Merck KGaA (Darmstadt, Germany) and Sigma‐Aldrich (St. Louis, MO, USA). Microbiological media were sourced from Oxoid Ltd. (Basingstoke, UK) as specified in Section 2.7.

### 2.2 Sample Preparation

Freeze‐killed third‐instar larvae of *R. phoenicis* (mean weight: 8.2 ± 1.3 g per larva; mean length: 5.4 ± 0.8 cm) were obtained from the test laboratory of the CSIR‐Food Research Institute (Accra, Ghana). The larvae originated from a single commercial rearing batch sourced from a palm weevil farm in Kumasi, Ghana, where larvae were reared generally on oil palm ( *Elaeis guineensis* ) frond substrate under controlled conditions. Third‐instar was confirmed by morphological examination (body size, cuticle color, and head capsule width). Freeze‐killing at −20°C for 24 h and subsequent slitting (0.50 cm) were carried out before use (Figure 1).

**FIGURE 1:**
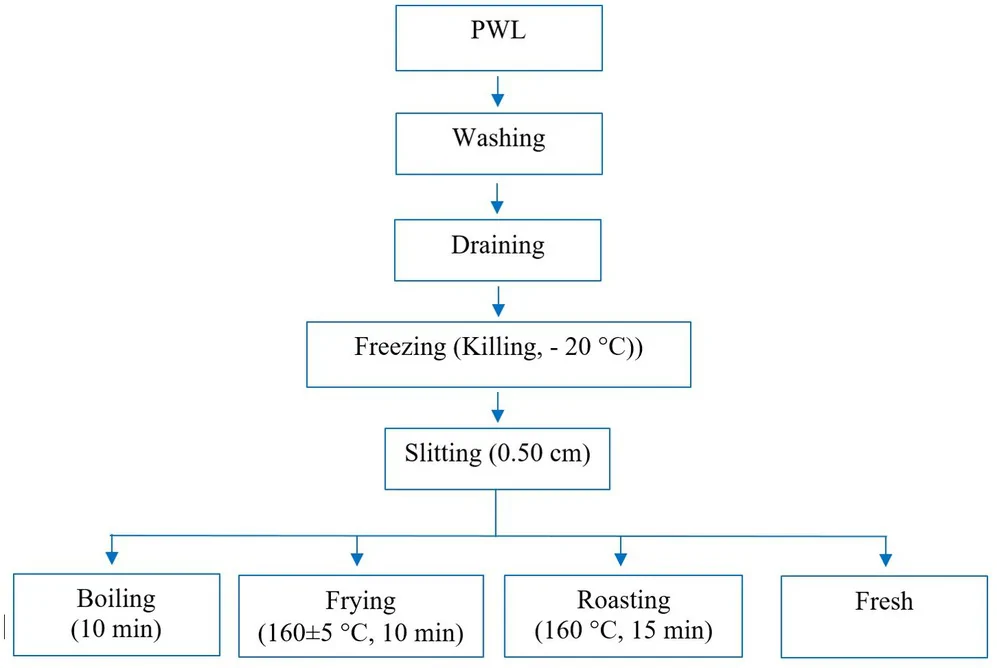
Process flow—sample preparation; PWL—Palm weevil larvae.

### 2.3 Proximate and Physicochemical Analyses

Moisture, protein (Kjeldahl), fat, ash, carbohydrate, and calories were determined using the AOAC International (2005) approach. Specifically, moisture by oven drying at 105°C to constant weight (AOAC 934.01); crude protein by Kjeldahl digestion and distillation with nitrogen‐to‐protein conversion factor of 6.25 (AOAC 2001.11); crude fat by Soxhlet petroleum ether extraction (AOAC 920.39); ash by muffle furnace combustion at 550°C for 4 h (AOAC 942.05); total carbohydrate by difference [100 − (moisture + ash + fat + protein) g/100 g]; energy calculated using Atwater conversion factors: 4 kcal/g for protein, 4 kcal/g carbohydrate, and 9 kcal/g for fat.

### 2.4 Water Activity (*a*_w_)

The *a*_w_ of the samples was determined using the Hygrolab *a*_w_ meter (Rotronic AG, Bassersdorf). Samples were equilibrated to 25°C ± 0.5°C in the measurement chamber prior to reading, as 25°C is the standard reference temperature for characterization of food and comparison with published values and regulatory limits. The sample readings were recorded directly.

### 2.5 Mineral/Heavy Metal Analyses

A 1.00 g sample was digested with 5 mL HNO_3_ and 3 mL H_2_O_2_ (170°C, 1000 watts, 50 bar, 1 h) respectively. After cooling, the resultant was diluted with deionized water to the 25 mL mark. The quantification was done using a calibration curve (*r* ^2^ > 0.99) of the corresponding standards for the elements. The calibration standard solutions of 0.5 mg/L, 1.0 mg/L, 2.0 mg/L and 4.0 mg/L for Cu, Mn and Fe; 0.2 mg/L, 0.4 mg/L and 0.8 mg/L for Zn; and 2.0 mg/L, 4.0 mg/L and 6.0 mg/L for Ca were prepared from a multielement standard stock solution by serial dilution. Mn, Ca, Cu, Fe, Zn were analyzed using a 240FS Varian AAS. The British standard test method BS EN 14084:2003 was used. Pb, Cd, Hg, and As were determined by Inductively Coupled Plasma Mass Spectrometer (ICP‐MS; Agilent 7700 Series). Also, one gram (1 g) of homogenized sample was transferred into a Teflon container, and 5 mL of nitric acid (HNO_3_) and 3 mL of hydrogen peroxide (H_2_O_2_) were added, tightly covered, and subjected to complete microwave (wet) digestion—170°C, 50 bar, 45 min. After allowing to cool (30 min), samples were transferred into 50 mL centrifuge tubes and topped up to the 25‐mL mark with deionized water for quantification. A certified reference material (CRM; Dorm 4 and a sample blank [prepared using distilled water]) were added to each batch of samples. Working standard Pb, Cd, Hg, and As solutions were prepared from their respective stock standard solutions of 1000 mg/L (ppm) by serial dilution (Table S1). The analysis mode of the auxiliary, plasma, helium, and carrier gases was 0–1.0 L/min, 15 L/min, 3–5 and 10–12 mL/min, and 0.8–1.3 L/min, respectively. The parameters for the forward power, Ar gas tank pressure, inlet and exhaust temperature, reflected power, plasma frequency, and detector were 700–1600 W, 500–700 kPa, 15–45 and 45–55 rpt °C, < 20 W, 26.0–28.0 MHz, and single mass spectroscopy, respectively. The accuracy of the method was evaluated by analyzing a CRM (Dorm 4‐Fish protein Certified Reference Material for Trace Metals and Other Constituents) alongside the prepared samples. The CRM was subjected to the same digestion procedure as the samples, prior to the metal (As, Hg, Pb, and Cd) analysis. The percentage recovery of the CRM and the coefficient of regression of the calibration curves (≤ 0.995) of the study was indicative of good analytical work. The instrument limit of detection (mg/L) of the analytes (Pb, Cd, Hg, and As) were 0.003, 0.003, 0.007, and 0.017, respectively; and that for quantification (mg/L) were 0.010, 0.001, 0.020, and 0.050, respectively. The sample‐weight‐based limits of detection (LOD), accounting for the dilution factor (sample weight: 1 g; final volume: 25 mL; dilution factor = 25), are: Pb: 0.075 mg/kg; Cd: 0.075 mg/kg; Hg: 0.175 mg/kg; As: 0.425 mg/kg. For values below the limit of detection, the standard LOD/2 substitution method was applied as recommended for censored environmental data (Helsel 2012; European Commission 2010).

### 2.6 Health Risk of Exposure to Heavy Metals Through Raw and Processed PWL

Potential health risk (non‐carcinogenic) associated with exposure to heavy metals was estimated using daily intake (EDI) (Vrhovnik et al. 2013), and target hazard quotient (THQ) developed by the United States (USA) Environmental Protection Agency (EPA) for the estimation of plausible health risks linked to long term exposure to chemicals/chemical pollutants (US EPA 2022; Wang et al. 2021). The daily amount consumed, body weight, and heavy metal concentrations in PWL samples are stated as follows:

EDI=C×IR/BW.

where C = mean concentration of heavy metal in PWL (mg/kg), IR = ingestion rate of PWL (assumed 0.05 kg/day), BW = average body weight of adults (assumed 70 kg).

Subsequently, the potential non‐carcinogenic risk of long‐term exposure to the contaminant under study (Hg, As, Pb, and Cd) in the PWL samples was estimated using THQ, computed as follows:

THQ=EDI/RfD.

where RfD = reference dose (mg/kg/day), from the US EPA or WHO; THQ < 1.00 implies Acceptable risk; THQ ≥ 1.00 implies a potential health concern.

For lead, which lacks a US EPA RfD due to the conclusion that no safe threshold exists for neurodevelopmental effects (US EPA 2022), the Margin of Exposure (MOE) framework was applied following EFSA guidance (EFSA [European Food Safety Authority] 2010):

$$\mathrm{MOE}={\text{BMDL}}_{01}/\mathrm{EDI}.$$

where BMDL_01_ for Pb = 0.50 μg/kg bw/day (= 0.0005 mg/kg bw/day; EFSA 2010). Following EFSA guidance for substances with genotoxic/carcinogenic potential, an MOE ≥ 10 is generally considered indicative of low health concern, whereas values below this threshold indicate exposure of potential concern.

For carcinogenic risk assessment of Cd (IARC Group 1 human carcinogen), the Incremental Lifetime Cancer Risk (ILCR) was calculated as follows:

ILCR=EDI×CSF.

where CSF (oral cancer slope factor for Cd) = 6.1 × 10^−3^ (kg·day/mg) per US EPA IRIS (US EPA 2022). An ILCR < 1 × 10^−6^ is considered acceptable, 1 × 10^−6^ to 1 × 10^−4^ is indicative of a risk that warrants consideration, and > 1 × 10^−4^ is considered high risk.

### 2.7 Microbiological Analyses

Ten grams each of the PWL samples (raw, cleaned, boiled, fried, and roasted) were weighed and homogenized in 90 mL of sterile diluent (0.8% Saline, 0.1% Peptone Solution, 7.2 pH) in a stomacher (Lab Blender, Model 4001, Seward Medical, London, England) at medium speed for 30 s. Tenfold serial dilutions were made with the diluents, and 1 mL or 0.1 mL of each diluted sample was pour‐plated or spread‐plated on different media for enumeration or detection. Aerobic mesophiles were enumerated on Plate Count Agar (Oxoid CM 325) incubated at 37°C for 48 h to 72 h in accordance with NMKL. No. 86 (2013), Yeast and molds were enumerated on Dichloran‐Rose Bengal Chloramphenicol Agar (Oxoid CM 0727) incubated at 25°C for 3–5 days in accordance with ISO 21527‐1:2008. *Enterobacteriaceae* were enumerated on Tryptone Soya agar (Oxoid CM 0131) incubated at 25°C for 2 h and overlaid with Violet Red Bile Glucose Agar and incubated at 37°C for 24 h in accordance with NMKL 144 (2005). *Staphylococcus aureus* was enumerated by spread plate on Bird Parker media (Oxoid CM 0275) supplemented with Egg Yolk Tellurite (SR54) and incubated at 37°C for 24 ± 3 h in accordance with NMKL No. 66, 4th edition (2009). *Clostridium perfringens* was enumerated on Perfringens agar base (Oxoid CM 015) and incubated under anaerobic conditions at 37°C for 22 h in accordance with ISO 7937 (2004(E)).

### 2.8 Spectral Analysis

Pulverized samples were dissolved in 0.01 mol/L (pH 8.0) buffer solution (phosphate‐PBS) to 1.5 mg/mL. Subsequently, the UV‐V spectra of the respective mixtures/solutions were examined (at the range 200–400 nm) using a UV‐V Dual‐beam Spectrophotometer (Shimadzu Corporation, UV‐1800, 240 V IVDD, USA), at 25°C. A 60 nm/min scan rate and 2.0 nm band‐width settings were applied. For the blank spectrum, the PBS solution was used.

### 2.9 Statistical Analyses

Proximate and mineral analyses were conducted on three independent analytical replicates (*n* = 3) per sample type. Each replicate was prepared from a homogenized composite of approximately 20 individual larvae to account for inter‐individual variability within the batch. Microbiological analyses were performed in duplicate (*n* = 2) per sample. Statistical analysis (one‐way ANOVA, Tukey's post hoc, *p* < 0.05) was performed on all data sets using Minitab 16.0. Inter‐batch variability was not assessed as all samples originated from a single commercial batch, which is acknowledged as a study limitation (see Sections 3.2 and 4).

## 3 Results and Discussion

### 3.1 Proximate Composition and Physicochemical Properties of Raw and Processed PWL

Boiling, frying, and roasting are important global and handy processing techniques that have been used in the past several decades to cook a wide spectrum of foods/products. Table 1 shows the proximal composition of the four (4) PWL samples considered in this study.

**TABLE 1:** Proximal composition of the four PWL samples.

Parameter (g/100 g)	Samples			
	Bo‐PWL	Fd‐PWL	Ro‐PWL	F‐PWL
Moisture	72.59 ± 0.26^a^	30.51 ± 0.27^d^	46.21 ± 0.29^c^	71.01 ± 0.17^b^
Ash	1.01 ± 0.12^c^	1.92 ± 0.15^a^	1.53 ± 0.12^ab^	1.14 ± 0.03^bc^
Fat	13.55 ± 0.09^c^	34.39 ± 0.18^a^	10.49 ± 0.09^d^	14.06 ± 0.05^b^
Protein	10.68 ± 0.09^d^	27.49 ± 0.04^a^	25.31 ± 0.09^b^	11.78 ± 0.09^c^
CHO	2.16 ± 0.56^c^	5.68 ± 0.64^b^	16.45 ± 0.59^a^	2.01 ± 0.34^c^
Energy	173.35 ± 1.02^d^	442.19 ± 0.78^a^	261.45 ± 1.14^b^	181.70. ± 0.55^c^
*a*_w_	0.77 ± 0.00^a^	0.73 ± 0.00^c^	0.72 ± 0.01^c^	0.76 ± 0.00^b^

*Note:* Different supercript letters means significantly different (*p* < 0.05).

Abbreviations: *a*_w_, water activity; Bo‐PWL, boiled palm weevil larvae; CHO, total carbohydrate; F‐PWL, fresh; Fd‐PWL, fried; Ro‐PWL, roasted.

With the exception of the boiled PWL, moisture content (%) decreased from 71.01% in the raw PWL to 30.51% in the fried and 46.21% in the roasted samples (*p* < 0.05). Thus, frying resulted in the highest moisture loss, while boiling slightly increased the moisture. Also, protein content increased from 11.78% in the raw PWL to 27.49% and 25.315% (*p* < 0.05) in the fried and roasted samples, respectively. The unique contribution of frying to sensory attributes of foods, as well as its relatively low cost of usage even at the industrial‐scale level, has made fried foods one of the staples of the growing fast food industry worldwide. As expected, all cooked PWL in this study showed a decrease in crude fat contents except for the fried (Table 1). Variations in moisture, fat, and protein content of other food matrices have been reported by other researchers also (Nzewi and Anthony 2011; Oboh et al. 2010). The observed variations in moisture, fat, and protein content of the treated PWL (boiled, fried, and roasted) samples (in this study) may be ascribed to differences in the operational‐mechanism of the cooking methods. Relative to water (H_2_O), the boiling point (BP) of oil is high (> 120°C). The BP of H_2_O is 100°C, and since that of hot oil (likewise hot air/dry heat with regard to roasting) is above 100°C, it may have vaporized the H_2_O in the PWL into steam, removing a significant amount of water from the PWL (i.e., a mechanism that makes fried/roasted foods crunchy/crispy). Again, since the BP of H_2_O is not as high as oil, if foods (e.g., PWL) are “fried” in H_2_O, they become soggy. And this could illustrate why the moisture content increased by 2.23%, and decreased by 57.03% and 34.92% for the boiled, fried and roasted samples, respectively, relative to the raw sample. The reduction in crude protein content from 11.78% (F‐PWL) to 10.68% (Bo‐PWL, Table 1) is consistent with what has been reported by other researchers for other animal food (fish) samples (Hainida and Ikram 2015), and this could be explained by potential leaching and/or drippings from the food sample. The observed proximal data in this study suggest that frying and roasting had a beneficial effect on the protein content of PWL.

Regarding the energy value (Table 1), increases were observed in the order Fd‐PWL > Ro‐PWL > F‐PWL > Bo‐PWL. This may be attributed to the fact that during frying, PWL absorbed some of the oil, as demonstrated by the increased fat content realized in this study (for the fried PWL). Thus, the absorbed oil may have contributed to increasing the caloric content of the fried sample, with reference to the remaining samples. Although the moisture content of the fried (Fd‐PWL) was lower than the roasted (Ro‐PWL), the *a*_w_ for these two samples was in the reverse, suggesting the relationship between moisture content and *a*_w_ of the said samples is not linear, consistent with literature (Juarez‐Enriquez et al. 2019).

### 3.2 Essential Mineral and Heavy Metal Content of Raw and Processed PWL

Minerals, in their simplest form, are inorganic elements obtained from soil. Though they are not used as an energy source, minerals (based on individual characteristics) play a vital role in the physiology of humans. Since humans cannot synthesize their own minerals, their daily mineral requirements must come from diets—plants or animal products (not overlooking water). As the mineral content of soil and/or water varies from one region to another, the mineral content of foods/water is also expected to be geographically different. Generally, minerals are well absorbed from animal‐based food products. Minerals are classified as either major minerals or trace minerals, depending on the amount needed in the body (Martinez‐finley et al. 2012). In this work, essential minerals—major (Ca) and trace minerals/essential metals (Fe, Cu, Zn, and Mn) content of PWL subjected to different cooking methods (boiling, frying, and roasting, compared to the raw sample) were considered.

The predominant minerals in all samples were Ca and Fe, ranging from 80.01–142.87 and 23.87–52.40 ppm, respectively (Table 2). The study results revealed that roasting and frying caused a significant increase (*p* < 0.05) in the Ca content, relative to the raw sample. The observed increase was in the order: Fd‐PWL > Ro‐PWL > F‐PWL (*p* < 0.05, Table 2). Thus, boiling had the least retention of Ca.

**TABLE 2:** Essential minerals/metals and heavy metals content of PWL subjected to different culinary treatment.

Parameter (ppm)	Samples			
	Bo‐PWL	F‐PWL	Ro‐PWL	Fd‐PWL
Ca	80.01 ± 0.21^c^	81.77 ± 1.83^c^	120.65 ± 0.08^b^	142.87 ± 0.04^a^
Zn	12.58 ± 1.04^ab^	14.04 ± 0.11^a^	9.97 ± 0.01^b^	10.61 ± 0.36^b^
Fe	23.87 ± 0.22^c^	24.12 ± 0.34^c^	45.01 ± 0.01^b^	52.40 ± 0.70^a^
Mn	5.14 ± 0.12^c^	5.27 ± 0.16^c^	9.82 ± 0.17^b^	11.68 ± 0.16^a^
Cu	2.59 ± 0.02^c^	2.68 ± 0.13^c^	4.37 ± 0.06^b^	5.18 ± 0.12^a^
Hg	< 0.175^a^	< 0.175^a^	< 0.175^a^	< 0.175^a^
As	< 0.425^a^	< 0.425^a^	< 0.425^a^	< 0.425^a^
Pb	0.3305 ± 0.0004^b^	0.3342 ± 0.0028^b^	0.3910 ± 0.0007^a^	0.39045 ± 0.0005^a^
Cd	0.8205 ± 0.0005^d^	0.8296 ± 0.0003^c^	0.9426 ± 0.0011^a^	0.93115 ± 0.0008^b^

*Note:* Means with the same superscript alphabets (in same row) are not significantly different from each other at *p* ≤ 0.05; < = below the limit of detection—Hg < LOD (0.175 mg/kg, sample basis) and As < LOD (0.425 mg/kg, sample basis); concentrations were substituted with LOD/2 for risk calculations in table 3 (Helsel 2012).

Abbreviations: Bo‐PWL, boiled palm weevil larvae; F‐PWL, fresh/raw palm weevil larvae; Fd‐PWL, fried palm weevil larvae; Ro‐PWL, roasted palm weevil larvae.

The fresh sample (as well as the boiled) retained more Zn than the roasted and fried samples (*p* < 0.05). This could be linked to the significant loss in moisture following the treatment, hence reducing the content of the said mineral, suggesting that some minerals may have leached with the loss of moisture during the cooking process. That said, literature shows disagreement in how cooking methods impact the mineral contents of various foods/food products (Bassey et al. 2014; Gokoglu et al. 2004). Some researchers, for example, report that boiling retains more minerals (iron and copper), whereas frying retains more zinc for some tubers (Irish/and sweet potato). The discrepancies in the retention of minerals in the literature information and the findings from this study may be due to the differences in the food matrices. It also follows that the method used in cooking PWL can alter the nutritional composition; thus, some cooking methods (in this regard) may be better for augmenting the nutritional (essential mineral) value than others (i.e., also applicable to the proximal composition of the PWL, too). Fe increased significantly when the frying method was used, followed by roasting (*p* < 0.05). Further, the mineral analysis revealed the PWL samples had moderate levels of Cu and Mn (2.59–5.18 and 5.14–11.68 ppm, respectively), which could be attributed to the feed used in raising the larvae.

Nonetheless, the chemical safety of edible insects (PWL) is of critical concern, especially regarding heavy (non‐essential/toxic) metals. Heavy metals such as Pb, As, Cd, and Hg (Scutaras and Carmen 2023) can accumulate in insects through their diet or environment. These metals could pose potential health risks to both the insects themselves and consumers. For example, humans could be deprived of the full benefits of essential metals in food due to the presence of toxic metals, which could hijack the transporters of essential metals, making it impossible for human cells to acquire the nutrients (essential metals) from the extracellular environment (Martinez‐finley et al. 2012). The risk of death and/or chronic illness is also plausible when high doses of the toxic metals (in PWL) are consumed.

That said, the analysis revealed that Hg and As concentrations in the PWL samples were below the analytical method's limit of detection, on a dilution‐corrected sample basis (Hg < 0.175 mg/kg; As < 0.425 mg/kg), which may be attributed to the feed and/or environment in which the larvae were farmed. However, since the sample‐basis LOD for both metals exceeds the respective FAO/WHO Codex maximum tolerable limits (0.02 mg/kg for Hg; 0.01 mg/kg for As; FAO/WHO, 2011), the analytical method used could not confirm compliance with these limits; a more sensitive method would be required to verify that Hg and As levels in PWL are indeed below the regulatory thresholds. This uncertainty, together with the possibility of long‐term consumer exposure, warrants cautious interpretation of the Hg/As safety of PWL. The concentration of Pb in the PWL samples, however, exceeded (slightly) the 0.30 mg/kg MPL (maximum permissible limit) established by FAO/WHO Codex Alimentarius (FAO/WHO, 2011). Neurotoxic effects, particularly in children, and kidney problems in adults have been linked to long‐term exposure to Pb in other food matrices (Hsu et al. 2026), making this an imperative note for consumers. Likewise, the Cd concentration in all the samples exceeded the tolerable limit of 0.05 mg/kg (FAO/WHO, 2011). This may also pose a health risk/concern, with reference to long‐term exposures, since it could cause bone demineralization and kidney damage (Godt et al. 2006), assessing health risk for such exposures imperative.

On the subject, our results on the mineral (and proximal) profiles of PWL suggest it has the potential to meet the nutritional needs of many, especially in the context of locally sourced/traditional diets. In agreement with other study reports, it could be inferred that PWL represents a good source of Ca, Fe, Zn, and fat (Adobeze and Enemor 2022; Alamu et al. 2013). Although these findings suggest the potential of PWL in the fight against under‐nutrition, it is imperative to note that the nutrient content of the larvae (likewise edible insects in general) is subject to significant variation, which may be linked to differences in factors such as processing/cooking methods and also geographical differences, feed, lifecycle stage, etc. may apply.

That said, this study was based on a single commercial rearing batch from one farm in Kumasi, Ghana. The nutritional and heavy metal profiles of *R. phoenicis* are known to vary with rearing substrate, geographical origin, season, and larval developmental stage (Riekkinen et al. 2022; Oonincx et al. 2025). Our results should therefore be interpreted as characterizing the specific farmed batch studied, and generalization to wild‐harvested or other commercially farmed populations should be made with caution.

### 3.3 Health Risk of Exposure to Heavy Metals Through Raw and Processed PWL

The quantitative risk assessment data (Table 3) offers an important look at the potential non‐carcinogenic health risks linked to consuming 4 different PWL samples. The analysis follows established US EPA guidelines and zeroes in on 4 significant heavy metals. The findings showed that the PWL contains at least 2 heavy metals that exceed international safety standards, suggesting a potential health risk through long‐term exposure. This stresses the importance of continuous monitoring and provision of risk‐based consumption education, especially for the fact that PWL could become a source of dietary protein in targeted regions. Table 3 shows the EDI and THQ for the PWL samples, and the reference doses for the metals. THQ levels for the metals (Hg, As, Pb, and Cd) in the PWL samples are all < 1, indicating low non‐carcinogenic health risk from the metals. That notwithstanding, THQ showing Cd > 0.5 and As ≈0.5 (based on the LOD/2 substitution) are indicators that these metals could potentially approach/exceed the threshold of 1 if preventative measures (following root cause analysis) are not put in place; noting that the As THQ is derived from a substituted non‐detect value and should be interpreted with the same caution as the underlying LOD discussed above. In other words, it is key to consider these findings in a broader context rather than just focusing on the risks posed by individual metals. People are usually exposed to a mix of different contaminants, and the effects can add up.

**TABLE 3:** EDI, THQ, MOE, and ILCR for PWL samples, and reference doses for heavy metals.

Sample	Metal	Conc. (mg/kg)	EDI (mg/kg/day)	THQ	RfD (mg/kg/day)	Source	MOE	ILCR
Bo‐PWL	Hg (met.)	0.0875	0.0000625	0.6250	0.0001	US EPA (IRIS, US EPA 2022)	—	—
	As (iAs)	0.2125	0.0001518	0.5060	0.0003	US EPA	—	—
	Pb	0.3305	0.0002361	—	—	—	1.96	—
	Cd	0.8205	0.0005860	0.5860	0.001	US EPA	—	3.4 × 10^−6^
Fd‐PWL	Hg (met.)	0.0875	0.0000625	0.6250	Idem	Idem	—	—
	As (iAs)	0.2125	0.0001518	0.5060	Idem	Idem	—	—
	Pb	0.39045	0.0002789	—	—	—	1.79	—
	Cd	0.93115	0.0006651	0.6651	Idem	Idem	—	4.1 × 10^−6^
Ro‐PWL	Hg (met.)	0.0875	0.0000625	0.6250	Idem	Idem	—	—
	As (iAs)	0.2125	0.0001518	0.5060	Idem	Idem	—	—
	Pb	0.3910	0.0002793	—	—	—	1.79	—
	Cd	0.9426	0.0006733	0.6733	Idem	Idem	—	4.1 × 10^−6^
F‐PWL	Hg (met.)	0.0875	0.0000625	0.6250	Idem	Idem	—	—
	As (iAs)	0.2125	0.0001518	0.5060	Idem	Idem	—	—
	Pb	0.3342	0.0002387	—	—	—	1.93	—
	Cd	0.8296	0.0005926	0.5926	Idem	Idem	—	3.6 × 10^−6^

Abbreviations: Bo‐PWL, boiled palm weevil larvae; Conc, concentration; F‐PWL, fresh/raw palm weevil larvae; Fd‐PWL, fried palm weevil larvae; iAs, inorganic As; ILCR, EDI × CSF where CSF for Cd = 6.1 × 10^−3^ (kg·day/mg) (US EPA 2022); Met, methyl; MOE, BMDL_01_/EDI where BMDL_01_ for Pb = 0.0005 mg/kg/day (EFSA 2010); RfD, reference dose; Ro‐PWL, roasted palm weevil larvae.

A sensitivity analysis was conducted to assess the impact of varying daily intake rates on THQ values for Pb and Cd (Table S2). At an IR of 10 g/day, THQ values for all metals remained well below 1. However, at IR ≥ 30 g/day, Cd THQ for fried and roasted samples ranged from 0.399 to 0.404 at 30 g/day and from 0.665 to 0.673 at 50 g/day, indicating that consumers with higher intake levels may be at increased risk. This finding supports the conservative use of 50 g/day as an upper‐bound estimate for risk screening purposes.

For Pb, the Margin of Exposure (MOE) framework was applied because the US EPA has withdrawn the oral RfD, concluding that there is no safe threshold for neurodevelopmental effects. The EFSA BMDL_01_ for Pb is 0.50 μg/kg bw/day (EFSA, 2010). The MOE values for all PWL samples ranged from 1.79 to 1.96 (Table 3), which are substantially below the generally recommended MOE of ≥ 10 for substances with genotoxic/carcinogenic potential, indicating that exposure from PWL consumption is within a range of concern under the EFSA framework.

For Cd, which is classified as a Group 1 human carcinogen by IARC, the Incremental Lifetime Cancer Risk (ILCR) was calculated. ILCR values ranged from 3.4 × 10^−6^ (Bo‐PWL) to 4.1 × 10^−6^ (Fd‐PWL) (Table 3). These values exceed the US EPA acceptable risk level of 1 × 10^−6^ but remain below the 1 × 10^−4^ level considered high risk. This suggests that while the carcinogenic risk from Cd in PWL is not negligible, it is within a range that warrants continued monitoring and risk management measures.

### 3.4 Microbiological Safety of Raw and Processed PWL

The microbiological safety of the PWL was determined by assaying for indicator microorganisms and pathogens, including Aerobic mesophiles (APC), yeast, mold, *Enterobacteriaceae, Staphylococcus aureus, and Clostridium perfringens*, which are relevant to the production of the insect larvae, and are indications of the hygienic conditions during processing, the safety, and shelf life of the palm weevil larvae. Table 4 presents the mean population (log_10_) of microorganisms recorded in raw palm weevil larvae that were washed and processed under different cooking conditions, that is, boiling, frying, and roasting. As observed, raw PWL recorded the highest count in aerobic mesophiles, yeast mold, and *Enterobacteriaceae* (log_10_ 7.0, 4.3, 3.0, and 5.2, respectively) at *p* ≤ 0.05. The raw‐washed PWL recorded fairly high counts for aerobic mesophiles, yeast mold, and *Enterobacteriaceae* with log_10_ counts of 5.2, 4.1, 3.0, and 3.0 at *p* ≤ 0.05, respectively. Generally, there were no counts for *S. aureus* and *C. perfringens* in all samples tested. It can be observed in Table 4 that washing the raw palm weevil larvae with running water without sanitizer reduced the microbial load for aerobic mesophiles and *Enterobacteriaceae* in the raw samples by two log cycles; however, no change was observed in the microbial population of yeast and mold in the raw washed palm weevil larvae (Table 4). Cooking the raw washed palm weevil larvae by boiling, frying, and roasting reduced the population of aerobic mesophiles by 2, 3, and 3.5 log cycles, respectively. Notwithstanding, no growth was observed in the rest of the indicator organisms tested. This indicates the effectiveness of temperature in reducing the microbial population in palm weevil processing; and this is consistent with fundamental concepts of using heat to control microbial activity. Washing the raw palm weevil larvae with a sanitizer followed by rinsing with potable water may further reduce the microbial population prior to cooking. The high counts observed in the raw samples may have resulted from poor production or, as expected, rearing conditions from the feed used in palm weevil larvae production; however, it can be controlled by effective cooking at the appropriate temperature and time as illustrated in this study.

**TABLE 4:** Microbial population of raw palm weevil larvae, washed and processed by boiling, frying, and roasting.

Sample	APC	Yeast	Mold	*Enterobacteria‐ceae*	*Staphylococcus aureus*	*Clostridium perfringens*
F‐PWL	(7.0 ± 0.01)^a^	(4.3 ± 0.01)^a^	(3.0 ± 0.01)^a^	(5.2 ± 0.01)^a^	nd	nd
F‐PWL (washed)	(5.2 ± 0.01)^b^	(4.1 ± 0.05)^b^	(3.0 ± 0.4)^a^	(3.0 ± 0.1)^b^	nd	nd
Bo‐PWL	(3.0 ± 0.03)^c^	nd	nd	nd	nd	nd
Fd‐PWL	(2.1 ± 0.05)^d^	nd	nd	nd	nd	nd
Ro‐PWL	(1.5 ± 0.2)^d^	nd	nd	nd	nd	nd

*Note:* Means with the same superscript alphabets (in same column) are not significantly different from each other at *p* ≤ 0.05.

Abbreviations: APC, aerobic plate count; Bo‐PWL, boiled palm weevil larvae; Fd‐PWL, fried palm weevil larvae; F‐PWL, fresh/raw palm weevil larvae; nd, not detected; Ro‐PWL, roasted palm weevil larvae.

When compared with regulatory thresholds, the raw unwashed F‐PWL APC count of 7.0 log_10_ CFU/g, observed in the current study, substantially exceeds the EU limit of 5 log_10_ CFU/g for ready‐to‐eat foods (EU Regulation (EC) No 2073/2005) and is comparable to reported counts in other raw edible insects (Grabowski and Klein 2017). Boiled, fried, and roasted samples (1.5–3.0 log_10_ CFU/g) are within acceptable limits for cooked meat and insect food products (EU: ≤ 5 log_10_ CFU/g). *S. aureus* and *C. perfringens* were not detected in any sample, meeting both Codex (CAC/RCP 1–1969, Rev. 4; Codex Alimentarius Commission 2003) and EU non‐detectable criteria for these pathogens in ready‐to‐eat products (Table S3). Another striking observation was that, although the moisture content for the Fd‐PWL was low (reference to Ro‐PWL), the results suggest that the actual moisture available for microbial activity was low for the Ro‐PWL, resulting in the low microbial load for this sample (Ro‐PWL).

### 3.5 UV–Vis Spectral Profile of Raw and Processed PWL

The UV‐VS was done to clarify/compare the differences between the 4 samples considered in the study (boiled, fried, roasted, and uncooked PWL). The profile highlights transitions associated with conjugated π‐electron systems, protein chromophores, lipid peroxidation products, and phenolic‐like compounds. These findings are crucial for understanding both the nutrient composition and impact of processing on the samples. The spectra of the PWL (Figure 2) show that each processing method resulted in distinct UV patterns, indicating changes in the chemical composition of the larvae. The notable absorption bands in the 200–400 nm range are typical of complex biological matrices filled with peptides, aromatic amino acids, fatty acids, and/or secondary metabolites. The 190–230 nm range (i.e., Deep UV) shows absorption bands that are linked to π → π* electronic transitions in peptide bonds and saturated fatty acids, suggesting a relatively high presence of proteins and lipid esters, which supports the idea that *R. phoenicis* is rich in both proteins and lipids. This finding aligns with earlier studies that reported crude protein levels ranging from 35% to 55% and total lipid contents between 25% and 45% in PWL (Ekpo and Onigbinde 2007; Zielińska et al. 2015).

**FIGURE 2:**
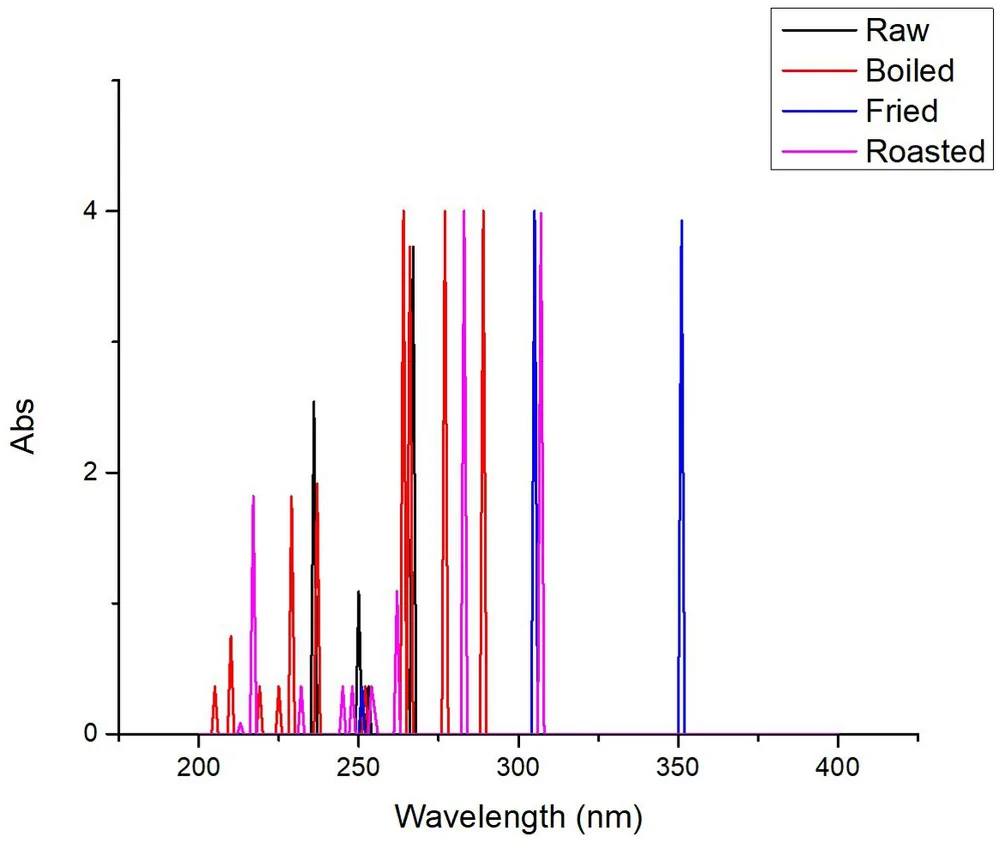
UV‐VS spectra of palm weevil larvae (PWL) samples subjected to different treatments.

Also, the absorption peaks for the raw and boiled samples were typically between 200 and 300 nm; and this may be ascribed to the high moisture content of both samples relative to the remaining samples. Consequently, the characteristic peak for the fried and roasted samples was in the higher wavelength—between 300 and 375 nm (although some peaks for the roasted sample were also found between 200 and 300 nm), suggesting alterations in protein structures. Also, the unique UV signatures of the fried and roasted larvae could be characteristic of Maillard reactions, indicative of protein and sugar interactions. The fried sample additionally had a single (stand‐alone) peak at 350 nm; and this could be due to lipid oxidation during the frying process and/or illustrated by the fact that the fried sample had the lowest moisture content among all the samples, which, as a consequence increased its fat content (Table 1). The findings suggest that the more moisture is removed from the PWL sample by a particular cooking method, the more distinct the spectral peaks become, with a shift to a higher wavelength (≈350 nm). In other words, samples with high moisture content are likely to have indistinct peaks at lower wavelengths (< 300 nm). Thus, the UV‐VS spectrum is a useful technique to illustrate the differences between PWL samples subjected to different cooking methods. From the peaks, it can be inferred that the samples were treated differently and thus their absorption peaks were different. Overall, the UV spectra provide valuable insights into the effects of processing (i.e., various heat treatments) on the composition of PWL.

### 3.6 Water Activity

Water activity is a critical parameter that influences the stability and safety of insect‐based food products. Monitoring the water activity of edible insects is essential to ensure their quality and shelf life related to pathogens in foods created the urgency for scientific investigations to understand the plausible causes and identify potential treatments to improve food safety. Thermal processing (different cooking methods) holds the potential to eliminate pathogens (e.g., *Salmonella*) in foods. And *a*_w_ is recognized as a basic factor influencing the thermal resistance of disease causing microorganisms in foods. Water activity is a thermodynamic property that varies significantly with temperature and dependent on the component of the product under consideration. The *a*_w_ for the PWL samples (raw/fresh—F‐PWL, fried—Fd‐PWL, roasted—Ro‐PWL, and boiled—Bo‐PWL) were in the order: Ro‐PWL < Fd‐PWL < F‐PWL < Bo‐PWL; and in the range 0.72–0.77. The difference between the fried and roasted samples was, however, not significant (*p* > 0.05). Meaning, both samples could, similarly, impede microbial activity/growth and thus their stability on the shelf may be similar. Thus, different cooking methods could impart the *a*_w_ of resultant PWL samples.

## 4 Conclusion

The proximal composition, mineral content, chemical safety, human health risk, microbiological quality, physicochemical characteristics, and UV–vis spectra of *R. phoenicis* larvae (PWL) samples (fresh, boiled, fried, and roasted) were examined. The moisture content of the fried and roasted samples was significantly reduced from 71.01 to 30.5 and 46.2%, respectively, but the protein content was increased by 133.36% and 114.89%, respectively, compared to the fresh larvae. The fat content of the fried sample (34.4%) was highest compared to the remaining samples and correlated to increased energy value—442.19 kcal/100 g. Calcium (Ca) and iron (Fe) contents increased significantly, subsequent to frying and roasting, but zinc was less retained in the same samples. Mercury and arsenic were not detected above the method's sample‐basis limits of detection (Hg < 0.175 mg/kg; As < 0.425 mg/kg) because these LODs exceed the respective Codex limits (0.02 mg/kg for Hg; 0.01 mg/kg for As); compliance with those limits could not be demonstrated. Lead and cadmium (0.33–0.39 and 0.82–0.94 mg/kg, respectively) exceeded Codex maximum limits. On health risk, THQ values for all metals were less than one (< 1), indicative of low non‐carcinogenic health risk. THQ values above 0.5 were observed for both Cd and As (the latter based on the LOD/2 substitution), which calls for immediate pre‐emptive actions to prevent these values from potentially exceeding one (1). ILCR values for cadmium (3.4–4.1 × 10^−6^) exceeded the US EPA acceptable level of 1 × 10^−6^, and MOE values for lead (1.79–1.96) indicated exposure is within a range of concern, warranting continued monitoring. The fresh PWL (washed or unwashed with water) was found to be microbiologically unsafe for human consumption, based on end‐product testing and comparison with EU regulatory thresholds. UV‐spectra data showed the processing methods used in treating the PWL samples were (each) unique, judging from distinctive UV patterns. It also affirmed that the PWL samples were typical complex biological matrices composed of peptides, aromatic amino acids, fatty acids, and/or secondary metabolites (in the 200–400 nm range). Further, samples with high moisture content were found to likely have indistinct peaks at lower wavelengths (< 300 nm).

The study outcomes provide valuable insights into the nutritional value and safety considerations of consuming farmed *R. phoenicis* from a commercial source in Ghana. This information can help policymakers, food safety authorities, and consumers make informed decisions regarding the inclusion of edible insects in the local diet and promote their sustainable use as a nutritious food source. Our findings, however, should be interpreted in line with the specific farmed batch studied, and generalization claims (to wild‐harvested or other commercially farmed larvae) should be made with caution. Future studies should incorporate multiple batches, farms, seasons, and regions to establish representative reference values. Further research and promotion efforts are also recommended to facilitate the integration of farmed *R. phoenicis* into local diets and promote their acceptance among potential or would‐be consumers. This is not a boundless study, and as such, our research team will continue to have systematic investigations on this novel food and/or associated products.

## Author Contributions

**Benjamin Kumah Mintah:** conceptualization, methodology, investigation, writing – original draft, writing – review and editing, formal analysis. **Hilary Kwesi Ketemepi:** investigation, methodology, writing – review and editing. **Ebenezer Asiamah:** investigation, methodology, writing – review and editing. **Akwasi Akomeah Agyekum:** investigation, methodology, formal analysis, writing – original draft, writing – review and editing. **Ethel Juliet Serwa Blessie:** investigation, methodology, writing – original draft. **Akua Boatemaa Arthur:** investigation, writing – original draft, methodology. **Mokhtar Dabbour:** methodology, writing – review and editing, formal analysis.

## Ethics Statement

This research did not involve any human or animal subjects; hence, ethical approval was not needed.

## Conflicts of Interest

The authors declare no conflicts of interest.

## Supporting information


**Table S1:** The preparation of working standards for the calibration curve.

**Table S2:** Sensitivity analysis: THQ values for Pb and Cd across a range of daily intake rates (IR = 10–50 g/day).

**Table S3:** Summary of regulatory thresholds and comparison with study findings.

## Data Availability

Data are available upon request.
