# Supplementary material for: Nutritional Composition, Chemical Safety, and Microbiological Quality of Farmed Palm Weevil Larvae (Rhynchophorus phoenicis) From a Commercial Rearing Unit in Ghana
Source: Food Sci Nutr. 2026 Sep 27;14(10):e72402. doi: 10.1002/fsn3.72402 (PMC13617201; doi:10.1002/fsn3.72402)
Supplement: Supplementary file 1 [file FSN3-14-e72402-s001.docx]

**Supplementary Table S2.** Sensitivity analysis: THQ values for Pb and Cd across a range of daily intake rates (IR = 10–50 g/day)

| Sample | IR (g/day) | Pb THQ* | Cd THQ |
| --- | --- | --- | --- |
| Bo-PWL | 10 | 0.013 | 0.117 |
| Bo-PWL | 20 | 0.026 | 0.234 |
| Bo-PWL | 30 | 0.039 | 0.351 |
| Bo-PWL | 50 | 0.066 | 0.586 |
| Fd-PWL | 10 | 0.016 | 0.133 |
| Fd-PWL | 20 | 0.031 | 0.266 |
| Fd-PWL | 30 | 0.047 | 0.399 |
| Fd-PWL | 50 | 0.078 | 0.665 |
| Ro-PWL | 10 | 0.016 | 0.135 |
| Ro-PWL | 20 | 0.031 | 0.269 |
| Ro-PWL | 30 | 0.047 | 0.404 |
| Ro-PWL | 50 | 0.078 | 0.673 |
| F-PWL | 10 | 0.013 | 0.119 |
| F-PWL | 20 | 0.027 | 0.237 |
| F-PWL | 30 | 0.040 | 0.356 |
| F-PWL | 50 | 0.066 | 0.593 |

*Note: THQ for Pb calculated using withdrawn US EPA RfD of 0.0036 mg/kg/day for comparative purposes only; health risk interpretation for Pb should use MOE framework.*
