# Supplementary material for: Nutritional Composition, Chemical Safety, and Microbiological Quality of Farmed Palm Weevil Larvae (Rhynchophorus phoenicis) From a Commercial Rearing Unit in Ghana
Source: Food Sci Nutr. 2026 Sep 27;14(10):e72402. doi: 10.1002/fsn3.72402 (PMC13617201; doi:10.1002/fsn3.72402)
Supplement: Supplementary file 2 [file FSN3-14-e72402-s002.docx]

**Table S3.** Summary of regulatory thresholds and comparison with study findings

| Parameter | Codex/EU Limit | F-PWL (raw) | Bo-PWL | Fd-PWL | Ro-PWL | Compliance |
| --- | --- | --- | --- | --- | --- | --- |
| APC (log CFU/g) | ≤5 (EU RTE) | 7.0 | 3.0 | 2.1 | 1.5 | Raw: non-compliant; Cooked: compliant |
| *S. aureus* | Not detectable | nd | nd | nd | nd | Compliant |
| *C. perfringens* | Not detectable | nd | nd | nd | nd | Compliant |
| Pb (mg/kg) | ≤0.30 (Codex) | 0.33 | 0.33 | 0.39 | 0.39 | Non-compliant |
| Cd (mg/kg) | ≤0.05 (Codex) | 0.83 | 0.82 | 0.93 | 0.94 | Non-compliant |
| Hg (mg/kg) | ≤0.02 | <0.175 | <0.175 | <0.175 | <0.175 | Compliance could not be demonstrated |
| As (mg/kg) | ≤0.01 | <0.425 | <0.425 | <0.425 | <0.425 | Compliance could not be demonstrated |

nd –non detected
