# Supplementary material for: Nutritional Composition, Chemical Safety, and Microbiological Quality of Farmed Palm Weevil Larvae (Rhynchophorus phoenicis) From a Commercial Rearing Unit in Ghana
Source: Food Sci Nutr. 2026 Sep 27;14(10):e72402. doi: 10.1002/fsn3.72402 (PMC13617201; doi:10.1002/fsn3.72402)
Supplement: Supplementary file 3 [file FSN3-14-e72402-s003.docx]

**Supplementary Table S1.** The preparation of working standards for the calibration curve

| Concentration of stock solution (ppb) | Volume of aliquot taken from the stock solution to prepare the working standards (mL) | Volume of the working standards prepared (mL) | Concentration of working standards prepared (ppb) |
| --- | --- | --- | --- |
| 1000000 | 1 | 100 | 10000 |
| 10000 | 1 | 100 | 100 |
| 10000 | 0.5 | 50 | 1 |
| 10000 | 2.5 | 50 | 5 |
| 10000 | 5.0 | 50 | 10 |
| 10000 | 10 | 50 | 20 |
| 10000 | 25 | 50 | 50 |
